# Type 3 Von Willebrand disease: two clinical cases of a rare disorder

**DOI:** 10.1093/omcr/omag037

**Published:** 2026-04-14

**Authors:** Khadija Belcadi Abassi, Karima Larbi Ouassou, Abdelilah Radi, Azzdine Laaraje, Sara Babour, Soukaina Ait Hmadouch, Amale Hassani, Mohammed E L Khorassani, Rachid Abilkassem

**Affiliations:** Department of Pediatrics, Mohammed V Military Instruction Hospital, Avenue des FAR, Rabat 10100, Morocco; Department of Pediatrics, Mohammed V Military Instruction Hospital, Avenue des FAR, Rabat 10100, Morocco; Department of Pediatrics, Mohammed V Military Instruction Hospital, Avenue des FAR, Rabat 10100, Morocco; Department of Pediatrics, Mohammed V Military Instruction Hospital, Avenue des FAR, Rabat 10100, Morocco; Department of Pediatrics, Mohammed V Military Instruction Hospital, Avenue des FAR, Rabat 10100, Morocco; Department of Pediatrics, Mohammed V Military Instruction Hospital, Avenue des FAR, Rabat 10100, Morocco; Department of Pediatrics, Mohammed V Military Instruction Hospital, Avenue des FAR, Rabat 10100, Morocco; Hemophilia and Inherited Bleeding Disorders Treatment Center, Pediatric Hemato-Oncology Unit, Children’s Hospital of Rabat, Rabat 10100, Morocco; Department of Pediatrics, Mohammed V Military Instruction Hospital, Avenue des FAR, Rabat 10100, Morocco

**Keywords:** type 3 Von Willebrand disease, coagulopathy, Hemorrhage

## Abstract

Von Willebrand disease (VWD) is the most common inherited coagulopathy, resulting from a deficiency of von Willebrand factor (VWF), which plays a central role in hemostasis. Type 3 VWD, the rarest and most severe form, is characterized by an almost complete absence of VWF, leading to severe and recurrent bleeding episodes. We report two pediatric cases of third-degree cousins, aged 3 and 2 years, born to consanguineous parents, who presented with severe hemorrhagic syndromes including profuse epistaxis, gingival bleeding, and anemia. Both had markedly prolonged activated partial thromboplastin time, reduced factor VIII, and severely diminished VWF activity and antigen levels, confirming the diagnosis of severe type 3 von Willebrand disease. Management included local hemostasis, avoidance of trauma, and prophylactic administration of plasma-derived VWF/FVIII before invasive procedures. These cases highlight the diagnostic and therapeutic challenges of managing severe inherited bleeding disorders in resource-limited settings, emphasizing the importance of early recognition, family education, and structured follow-up.

## Introduction

Von Willebrand disease (VWD) is the most common inherited bleeding disorder, first described in 1926 by Erik von Willebrand. It results from a quantitative or qualitative deficiency of von Willebrand factor (VWF), a glycoprotein essential for primary hemostasis and stabilization of factor VIII. Inheritance can be autosomal dominant or recessive, depending on the subtype [1].

Type 3 VWD is the rarest and most severe form, with an estimated prevalence of approximately 1 in 1 000 000 individuals [1]. It is characterized by an almost complete absence of VWF, resulting in severe hemorrhagic syndromes with spontaneous or post-traumatic bleeding episodes from early childhood. Diagnosis relies on specific laboratory findings, including markedly reduced VWF activity and antigen levels, prolonged activated partial thromboplastin time (aPTT), and decreased factor VIII levels [1, 2].

The management of type 3 VWD requires a multidisciplinary approach, including prophylactic replacement with plasma-derived VWF/FVIII concentrates, patient and family education, and long-term follow-up to prevent complications. Recent studies have emphasized the role of early diagnosis, structured prophylaxis, and comprehensive care in improving outcomes and quality of life in affected children [3, 4]. Through the analysis of two pediatric cases, we aim to highlight the diagnostic and therapeutic challenges of this rare and severe condition.

## Case report

We report two cases of third-degree cousins, aged 3 and 2 years, born to consanguineous parents. Both were admitted to our department for the etiological investigation of a hemorrhagic syndrome characterized by recurrent profuse epistaxis, significant gingival bleeding, and anemia.

Their medical histories revealed frequent spontaneous epistaxis since the age of one, easy bruising after minor trauma, and frequent mucosal bleeding. Neither patient had undergone surgery or any invasive procedure prior to admission. Previous bleeding episodes had been managed symptomatically, without thorough investigation or specialized follow-up.

On physical examination, both patients appeared slightly pale due to cumulative blood loss from recurrent epistaxis;Admission hemoglobin levels were 8.9 g/dl (patient 1) and 11.9 g/dl (patient 2), with no clinical signs of internal bleeding and no active bleeding at the time of admission. There was no hepatosplenomegaly, joint swelling, or hematoma. Given the severity and frequency of the bleeding episodes, an extensive coagulation workup was performed.

Table 1 summarizes their main biological and coagulation findings.

**Table 1:** Baseline biochemical and coagulation profiles of both patients.

Parameter	Patient 1 (3 years)	Patient 2 (2 years)
Hemoglobin (g/dl)	8.9	11.9
MCV (fl)	86	84
MCH (pg)	29	28
WBC (/mm^3^)	16 000	14 500
Neutrophils (/mm^3^)	6900	6200
Lymphocytes (/mm^3^)	4400	4100
Platelets (/mm^3^)	351 000	340 000
AST (U/l)	35	32
ALT (U/l)	10	9
Na^+^ (mmol/l)	135	136
K^+^ (mmol/l)	3.5	3.7
PT (%)	100	98
aPTT (ratio)	3	2.3
P TT (seconds)	90	69
Factor VIII (%)	2.2	4
Factor IX (%)	88	91
Factor XI (%)	71	69
Factor XII (%)	43	45
VWF activity (%)	3	1.1
VWF antigen (%)	9.9	1.9

These results confirmed type 3 von Willebrand disease in both patients, characterized by an almost complete deficiency of VWF and markedly low factor VIII levels.

In light of the high hemorrhagic risk, a targeted management plan was implemented, including:

- Local hemostasis for minor bleeding episodes.
- Avoidance of high-risk trauma situations.
- Prophylactic infusion of VWF/FVIII before any invasive procedure.
- Immediate consultation in case of major bleeding.

A disease identification card was provided to the families to ensure prompt and appropriate emergency care when needed.

A long-term prophylaxis program is being considered, involving regular infusions of plasma-derived VWF combined with factor VIII to reduce the frequency of bleeding episodes and prevent severe complications. This proactive approach aims to improve the patients' quality of life through structured follow-up and preventive care.

## Discussion

First described in 1926 by Erik von Willebrand in a family from the Åland Islands in Finland, von Willebrand disease (VWD) is the most common inherited bleeding disorder, with a prevalence ranging from 0.6% to 1.3% in the general population, surpassing hemophilia [3]. This hemorrhagic disorder, transmitted predominantly in an autosomal dominant manner, affects both sexes and results from a quantitative or qualitative defect of von Willebrand factor (VWF), a glycoprotein essential for platelet adhesion and stabilization of factor VIII within the coagulation cascade [3, 4].

Three types of hereditary deficiencies are recognized, each with variable clinical manifestations. Type 1 is the most common form, characterized by a partial quantitative reduction in plasma VWF, inherited in an autosomal dominant fashion. Type 2 involves qualitative defects of VWF and is further subdivided into subtypes 2A, 2B, 2 M, and 2 N, depending on the underlying mechanism. Type 3 VWD, the focus of this report, is the rarest and most severe form, usually inherited in an autosomal recessive manner, explaining its higher prevalence in consanguineous populations [5]. This form is characterized by a near-total absence of VWF, leading to severe bleeding manifestations from early childhood, including massive epistaxis, significant gingival bleeding, frequent bruising, and post-traumatic or postoperative hemorrhage [5].

The combined deficiency of VWF and FVIII:C results in a dual defect affecting both primary and secondary hemostasis, manifesting as severe mucosal bleeding and hemophilia-like symptoms such as hemarthrosis and muscle hematomas. In women, menorrhagia may be particularly disabling. Diagnosis is based on a combination: prolonged aPTT, markedly reduced VWF antigen and functional activity (<5%), and decreased factor VIII levels. The laboratory profile of type 3 VWD often resembles that of moderate to severe hemophilia A, due to the absence of VWF [6].

Therapeutic management involves the administration of VWF/FVIII concentrates during bleeding episodes and as prophylaxis in high-risk patients. Desmopressin (DDAVP), which is effective in mild to moderate forms, is ineffective in type 3 due to the total absence of VWF [7]. Regular prophylaxis is recommended for patients with recurrent or high-risk bleeding, reducing bleeding frequency, hospitalizations, and long-term complications such as arthropathy. Patient and family education is a key component of care, especially prior to invasive procedures, and requires coordination with a multidisciplinary team including hematologists, anesthesiologists, surgeons, and dentists [7].

In surgical settings, plasma levels of approximately 1.00 IU/ml of VWF and FVIII should be achieved preoperatively, and residual levels > 0.50 IU/ml should be maintained postoperatively [8]. A systematic review demonstrated that maintaining these targets for at least three days after major surgery ensured optimal hemostasis in 74% to 100% of cases [8].

The efficacy of prophylaxis is well established, similar to hemophilia management. A post hoc analysis involving 331 VWD patients demonstrated a significant reduction in bleeding episodes and chronic pain in patients receiving prophylaxis [7]. However, its use remains limited; a survey of 6208 patients found that only 1.6% were on prophylaxis, mostly those with type 3 disease and a history of hemarthrosis [8]. The WIL-31 study further confirmed the efficacy and safety of Wilate prophylaxis, with 2 to 3 low-dose infusions per week providing effective protection regardless of sex, age, or VWD type [9].

During treatment with combined VWF/FVIII concentrates, close monitoring of FVIII levels is essential, as its prolonged half-life may lead to accumulation and an increased risk of venous thrombosis. A rare but serious complication is the development of alloantibodies against VWF, occurring in approximately 5% to 10% of type 3 VWD patients, particularly those with complete or partial gene deletions [8]. In such cases, alternative therapeutic strategies include recombinant FVIII for surgical procedures, bypassing agents such as recombinant activated factor VII (rFVIIa), or emicizumab, a bispecific monoclonal antibody mimicking FVIII function, which has recently shown promising results [10].

For a better understanding of the disease spectrum, Table 2 presents the main clinical and laboratory differences among the three major types of von Willebrand disease.

**Table 2:** Comparative features of the main types of von Willebrand disease (VWD).

Feature	Type 1 (Partial quantitative deficiency)	Type 2 (Qualitative defect)	Type 3 (Virtually complete deficiency)
Inheritance pattern	Autosomal dominant (rarely recessive)	Usually autosomal dominant (except 2 N, recessive)	Autosomal recessive
Genetic basis	Heterozygous mutations causing reduced synthesis or secretion of vWF	Missense mutations altering vWF structure or function	Homozygous or compound heterozygous null mutations
Plasma vWF antigen (vWF:Ag)	Mild to moderate reduction (typically 20–50 IU/dl)	Normal or mildly reduced	Undetectable or extremely low (<5 IU/dl)
vWF activity (vWF:RCo, vWF:GPIbM, or vWF:CB)	Proportionate decrease to antigen	Disproportionately decreased relative to vWF:Ag	Absent
vWF multimer pattern	Normal	Abnormal — subtype dependent: 2A (loss of HMW multimers), 2B (gain of function), 2 M (defective GPIb binding), 2 N (reduced FVIII binding)	Absent
Factor VIII level (FVIII:C)	Normal or mildly reduced	Mild to moderate reduction (notably in type 2 N)	Markedly reduced (<10 IU/dl)
Bleeding phenotype	Mild to moderate mucocutaneous bleeding (epistaxis, bruising, menorrhagia)	Variable severity — may mimic type 1 or hemophilia A (2 N)	Severe mucocutaneous and deep bleeding (joint or muscle hemorrhages)
Response to DDAVP (desmopressin)	Usually good and predictable	Variable; contraindicated in type 2B due to thrombocytopenia risk	Ineffective
Preferred treatment	DDAVP for minor bleeding or procedures; vWF concentrate if refractory	vWF/FVIII concentrates for moderate/severe cases; avoid DDAVP in 2B	vWF/FVIII replacement concentrates ± prophylaxis
Diagnostic clues	Parallel fall of vWF:Ag and vWF activity; normal multimers		

## Conclusion

Type 3 von Willebrand disease is the rarest and most severe form of VWD, posing significant diagnostic and therapeutic challenges, particularly in resource-limited settings. Early diagnosis, personalized prophylaxis, and multidisciplinary care are essential to prevent life-threatening bleeding and improve long-term outcomes. Management relies on specific coagulation studies and replacement therapy with VWF/FVIII concentrates, with an increasing emphasis on prophylaxis. Continuous family education, regular follow-up, and close collaboration between clinicians, laboratories, and families are crucial to achieving comprehensive care and enhancing the quality of life of affected patients.

**Learning Points**

- Type 3 von Willebrand disease should be considered in children from consanguineous families presenting with recurrent mucosal bleeding and severe anemia.
- Early diagnosis and regular prophylactic replacement with plasma-derived VWF/FVIII are essential to prevent life-threatening hemorrhagic complications.
- Family education, genetic counseling, and structured long-term follow-up significantly improve clinical outcomes and quality of life in affected children.
